# Mutations in RASA1 and GDF2 identified in patients with clinical features of hereditary hemorrhagic telangiectasia

**DOI:** 10.1038/hgv.2015.40

**Published:** 2015-11-05

**Authors:** Felicia Hernandez, Robert Huether, Lester Carter, Tami Johnston, Jennifer Thompson, James R Gossage, Elizabeth Chao, Aaron M Elliott

**Affiliations:** 1 Department of Research and Development, Ambry Genetics, Aliso Viejo, CA, USA; 2 Department of Bioinformatics, Ambry Genetics, Aliso Viejo, CA, USA; 3 Department of Clinical Genetics, Ambry Genetics, Aliso Viejo, CA, USA; 4 Division of Pulmonary/Critical Care, Georgia Regents University, Augusta, GA, USA

## Abstract

Hereditary hemorrhagic telangiectasia (HHT) is an autosomal dominant vascular disorder caused by mutations in *ENG*, *ACVRL1* and *SMAD4*, which function in regulating the transforming growth factor beta and bone morphogenetic protein signaling pathways. Symptoms of HHT can be present in individuals who test negative for mutations in these three genes indicating other genes may be involved. In this study, we tested for mutations in two genes, *RASA1* and *GDF2*, which were recently reported to be involved in vascular disorders. To determine whether *RASA1* and *GDF2* have phenotypic overlap with HHT and should be included in diagnostic testing, we developed a next-generation sequencing assay to detect mutations in 93 unrelated individuals who previously tested negative for mutations in *ENG*, *ACVRL1* and *SMAD4*, but were clinically suspected to have HHT. Pathogenic mutations in *RASA1* were identified in two samples (2.15%) and a variant of unknown significance in *GDF2* was detected in one sample. All three individuals experienced epistaxis with dermal lesions described in medical records as telangiectases. These results indicate that the inclusion of *RASA1* and *GDF2* screening in individuals suspected to have HHT will increase the detection rate and aid clinicians in making an accurate diagnosis.

## Introduction

Hereditary hemorrhagic telangiectasia (HHT) is an autosomal dominant vascular disorder that affects 1 in 5,000–10,000 individuals. It is characterized by skin and nasal telangiectases along with arteriovenous malformations (AVMs) in the brain, lungs and liver.^1^ The telangiectatic lesions are punctate, radiating or arborizing and affected individuals generally present with hemorrhages from nasal mucosa.^1^ HHT is one of the most common vascular disorders and mutations in *ENG*, *ACVRL1(ALK)* and *SMAD4* result in HHT type 1, HHT type 2, and combined juvenile polyposis and HHT syndrome, respectively.^1^ When preselected for meeting at least 3 of 4 Curacao criteria (epistaxis, telangiectases, AVMs and family history), ~87% of HHT cases are associated with identifiable mutations in *ENG*, *ACVRL1* and SMAD4. No known molecular cause is attributed to the remaining ~13% of clinically suspected cases.^1,2^ However, of the 892 cases sent to Ambry Genetics from 2008 to 2014, where HHT was either indicated or suspected, the detection rate for causative mutations identified in *ENG*, *ACVRL1* and *SMAD4* was ~47%. These data suggest that HHT may result from mutations in genes that have yet to be discovered or that phenotypes between vascular disorders have significant overlap, making it difficult for the clinician to make an accurate diagnosis.

Heterozygous mutations in the *RASA1* gene have been described in families with capillary malformations (CMs) with or without AVMs and/or arteriovenous fistulas.^3,4^ The specific condition is termed CM–AVM and can be clinically distinguished from HHT upon expert examination. The telangiectases seen with CM–AVM are larger than those described in HHT, but are still typically <1 cm, homogenous and pink to red. In addition, ~30% of diagnosed cases of CM–AVM have AVMs in the skin, muscle, bones of the face, ears, thorax, extremities, brain and spine.^4^ In HHT, AVMs can also be present in the brain but differ from CM–AVM in that the liver and lungs can be affected as well.^4^ Although patients with *RASA1* mutations may be distinguishable upon careful examination, there is significant phenotypic overlap with HHT possibly confounding diagnosis.

Recently, mutations in the *GDF2* gene, which encodes the protein bone morphogenetic protein 9, have been reported in patients with symptoms similar to HHT.^5^ *GDF2* is also part of the transforming growth factor beta and bone morphogenetic protein signaling pathways and is a ligand for type 1 (*ACVRL1*) and type II cell surface receptors as well as the auxiliary receptor, *ENG*.^6^ Wooderchak-Donahue *et al.*^5^ identified three mutations in *GDF2* in a cohort of 191 unrelated individuals who were suspected to have HHT, but who tested negative for mutations in *ENG, ACVRL1,* and *SMAD4*. The individuals all suffered from epistaxis and had dermal lesions resembling those of telangiectases indicating a possible role for mutations within *GDF2* in vascular disorders.^5^

The complex genetic heterogeneity and the overlapping phenotypes between patients with classic HHT and those harboring *RASA1* and *GDF2* mutations may make it difficult for clinicians to accurately identify the molecular lesion underlying the clinical presentation. Currently, most diagnostic labs offering mutational analysis for HHT focus on analyzing only *ACVRL1*, *ENG* and *SMAD4*. To determine whether *RASA1* and *GDF2* should be included in HHT panel testing, we utilized next-generation sequencing (NGS) to test for *RASA1* and *GDF2* mutations in 93 patient samples clinically suspected to have HHT that had previously tested negative for mutations in *ENG, ACVRL1* and *SMAD4*.

## Materials and methods

### DNA samples

All samples used in the analysis were from patients suspected to have HHT who were referred to Ambry Genetics from January 2013 to May 2014 for comprehensive HHT testing, which includes full gene-sequencing analysis of *SMAD4*, *ACVRL1* and *ENG* genes, and deletion/duplication analysis of *ACVRL1* and *ENG* genes. Subjects were excluded from this protocol due to a lack of sufficient DNA or written consent. Of the 93 individuals tested, 5 met at least 3 of 4 Curacao criteria, 23 met 2 and 65 met 1 or less. All individuals used for testing provided written consent and were de-identified before analysis (Solutions Institutional Review Board protocol #1OCT14–93). Clinical and phenotype data were abstracted from the test requisition form, provided by the ordering physician. At least 6–7 μg of genomic DNA was extracted from whole blood or saliva using the QiaSymphony instrument (Qiagen, Hilden, Germany) according to the manufacturer’s instructions. Isolated DNA was quantified using a NanoDrop UV spectrophotometer (Thermo Fisher Scientific, Waltham, MA, USA) with quality metrics of A260/280 from 1.8 to 2.0 and A260/230 of ⩾1.6.

### Primer library design

Custom designed primer sets were used to amplify and tag PCR products for sequencing according to the manufacturer’s suggested parameters (Fluidigm, San Francisco, CA, USA). The adapter sequences, 5′-AATGATACGGCGACCACCGAGATCT-3′ (forward primer) and 5′-CAAGCAGAAGACGGCATACGAGAT-3′ (reverse primer), were added to the target-specific portion of the primer and correspond to a portion of the Illumina NGS adapter (Illumina, San Diego, CA, USA). The final library consisted of 85 amplicons ~150–200 base pairs (bp) in size and covered all coding exons of *RASA1* and *GDF2* with at least 50-bp flanking intronic sequences. Primers were designed to avoid placement on known single-nucleotide polymorphisms (dbSNP138) and pathogenic gene mutations.

### Target enrichment and NGS library preparation

A amount of 50 ng of genomic DNA per sample was used to generate targeted, microfluidic-amplified PCR products using Access Array Target-specific Primers according to the manufacturer's instructions (Fluidigm). Amplicons were collected and a secondary PCR was performed to attach sample barcodes and sequencing tags for sequence ready amplicons. The secondary PCR amplification was performed in a Bio-Rad MyCycler (Bio-Rad, Hercules, CA, USA) with the following conditions: 95 °C for 10 min, followed by a program of 95 °C for 15 s, 60 °C for 30 s and 72 °C for 60 s for 15 cycles and ending with a 3-min extension at 72 °C. Reactions were purified using AMPure XP beads (Beckman Coulter, Brea, CA, USA) according to the manufacturer’s instructions and analyzed using a Bioanalyzer instrument (Agilent Technologies, Santa Clara, CA, USA). The final libraries were ~250–350 bp in size. Libraries were diluted to 10 nmol l^−1^ and up to 48 samples pooled together for sequencing. Sequencing was conducted on the Illumina HiSeq2500 using 150 bp paired-end conditions as described in the manufacturer’s standard workflow (Illumina).

### NGS analysis parameters

Initial data processing and base calling were performed as previously described.^7^ Ambry Genetics’ custom NGS bioinformatics pipeline utilized Novoalign V3.00.05 (Novocraft Technologies, Selangor, Malaysia). The bioinformatic NGS pipeline filters variants with a Q score ⩾20 and an allele count ⩾50×, along with no/low coverage regions that are ⩾50× coverage. During variant calling, ‘primer trimming’ was performed as described in Chong *et al*.^7^ The newly trimmed BAM output file was used as an input for the subsequent variant calling by GATK V2013.1-2.4.9 (Broad Institute, Cambridge, MA, USA). Variants of each sample within the reportable range (coding exons plus at least 5 bases of flanking intronic sequence) were filtered and classified based on public and internal data sources.^7^ Sanger sequencing was used to confirm all identified variants.

### Variant classification

Variants were then classified based on a 5-Tier classification algorithm based on pathogenicity as previously described.^8^ Alterations were further analyzed using SIFT, PolyPhen-2, MutationTaster (data not shown) prediction modeling software and detailed structural analysis.^9–11^ A three-dimensional model of the PH domain of RASA1 was generated using Phyre server in normal mode.^12^ Residues 474–577 of (NP_002881) were modeled using the C-terminal PH domain of human pleckstrin (PDB 1XX0: amino acids 235–347) as a template (30% sequence identity).^13^ The generated model was high quality (99.8% confidence over 99% of the model according the Phyre model analysis) with good modeling statistics. Figures were generated with chimera and alignments with ESPript3.^14,15^ Variants were based on *RASA1* reference sequence NM_002890.2 and *GDF2* reference sequence NM_016204.1.

## Results

**Figure 1:**
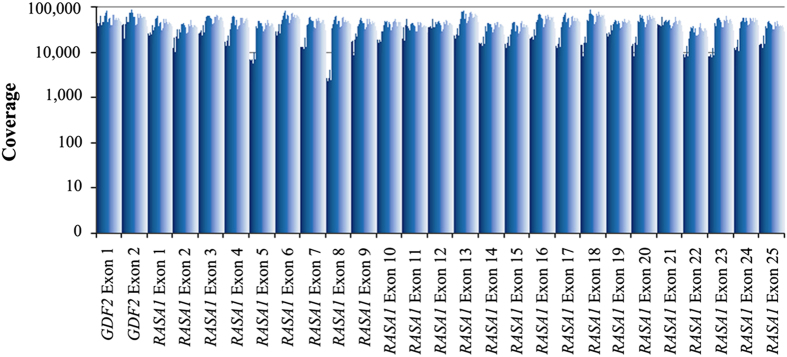
Average depth of sequencing coverage for each exon of *RASA1* and *GDF2* from 93 samples.

**Figure 2:**
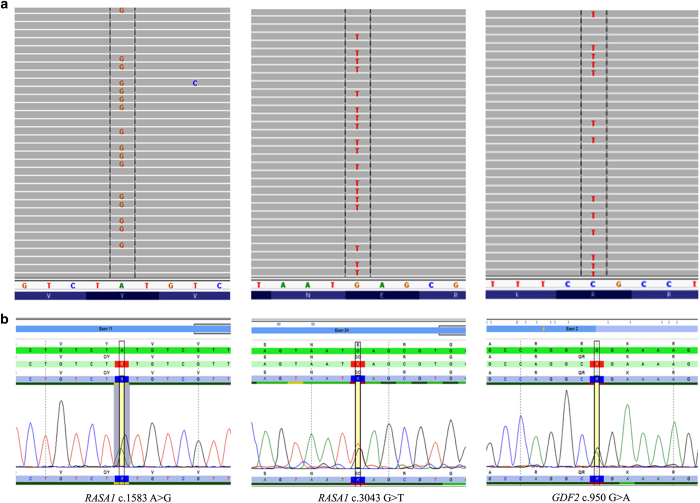
Potentially causative variants detected in *RASA1* and *GDF2* in samples suspected to have hereditary hemorrhagic telangiectasia (HHT). (**a**) Heterozygous mutations in *RASA1* and *GDF2* detected by NGS assay and (**b**) Sanger confirmed.

To determine whether including *RASA1* and/or *GDF2* in molecular testing for HHT increases diagnostic yield, the full coding regions of these two genes were analyzed from 93 patients suspected to have HHT, but tested negative for mutations in *ENG, ACVRL1* and *SMAD4*. A custom multiplex primer-based target enrichment assay coupled with NGS was utilized to detect variants in the patient samples. On average, 95% of the sequencing reads were on target, mapping specifically to the intended targeted regions. The mean read depth across all samples was extremely high at 33,166× (Figure 1). The data were processed through a custom bioinformatics pipeline, which utilizes several variant databases and protein prediction models. Results identified two pathogenic *RASA1* variants and a *GDF2* variant, which required further examination (Table 1, Figure 2). The presence of these variants in the samples was further confirmed by an independent sequencing methodology (Sanger; Figure 2).

The pathogenic *RASA1* c.1583A>G (p.Y528C) missense variant was located within the pleckstrin homology domain and was previously identified as a mutation in a family with CM–AVM.^3^ The population frequency of the variant is <0.1% in 1000 Genomes (5/5,003 alleles) and ESP (4/13,006 alleles) databases. The clinical features of the individual harboring the c.1583A>G mutation in our study were similar to HHT in that multiple blanching telangiectases on palms, fingers, tongue and toes were present. The nasal mucosa of the individual was reddened and epistaxis occurred almost daily. On the basis of the medical record, the individual met 2 of 4 Curacao criteria and investigation of visceral lesions was not performed. Both the father and son of the patient also had a history of epistaxis. In another individual, a novel *RASA1* nonsense variant, c.3043G>T (p.E1015*) located in the GTPase-activating protein domain (GAP) was detected. This variant has not been previously described in the literature and was not detected in the 1000 Genomes or ESP data sets. This individual met 2 of 4 Curacao criteria and initially presented during pregnancy with symptoms such as telangiectases along with epistaxis indicative of HHT. Furthermore, pulmonary findings showed shunting that was suspicious of pulmonary AVMs. However, upon evaluation of the lungs after pregnancy, results showed no pulmonary AVMs. The father and paternal aunt and uncle were noted as having telangiectases as well.

The *GDF2* c.950G>A (p.R317Q) missense variant identified was classified as having unknown significance (Table 1; Figure 2). *In silico* prediction tools, SIFT and POLYPHEN, were contradictory with variant classifications of damaging and benign, respectively. The variant has a very low population frequency of 0.05% determined by 1000 Genomes (1/2,098 alleles) and 0.01% (1/13,006 alleles) in the ESP data set. The patient was described by the clinician as having a definite diagnosis of HHT and test results for mutations in *ACVRL1*, *ENG* and *SMAD4* were negative. The patient met 3 of 4 Curacao criteria and had a history of nosebleeds, several telangiectases on fingers, lips and ears as well as pulmonary AVM. A suspicious family history was noted with two sons having frequent nose bleeds requiring cauterization as children but no AVMs.

All other variants detected in the samples were classified as benign polymorphisms. For *RASA1* these included c.296C>T (p.A99V), c.1454-7delT and c.1777-3delT. No additional variants in the analytical range for *GDF2* were identified.

## Discussion

The diagnosis of HHT is based on an examination of clinical features as well as molecular genetic testing. Currently, most diagnostic labs only screen for mutations in *ENG*, *ACVRL1* and *SMAD4* in patients suspected to have HHT. Our detection rate in these genes is ~47%, which is approximately half of what has been previously described in individuals who meet >3 Curacao criteria.^1^ This detection rate likely reflects the broader spectrum of patients sent for molecular testing including those who do not have a clinical HHT diagnosis, but for whom there is still sufficient suspicion based on clinical judgment. This suggests the involvement of other genes and/or significant overlap of HHT symptoms with other conditions.

Here we screened the *RASA1* and *GDF2* genes for mutations in 93 patients suspected to have HHT, but tested negative for *ENG*, *ACVRL1* and *SMAD4*, to determine whether their inclusion on an HHT testing panel would aid clinicians in making a diagnosis. We identified two patients with pathogenic *RASA1* mutations and one patient with a highly suspect *GDF2* variant of unknown significance. We cannot rule out the possibility that testing for gross deletions and duplications in *RASA1* and *GDF2* would have further increased the diagnostic yield.

**Figure 3:**
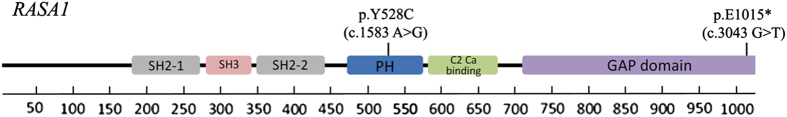
Protein domain structure of RASA1 depicting the location of the two pathogenic mutations identified in the study.

**Figure 4:**
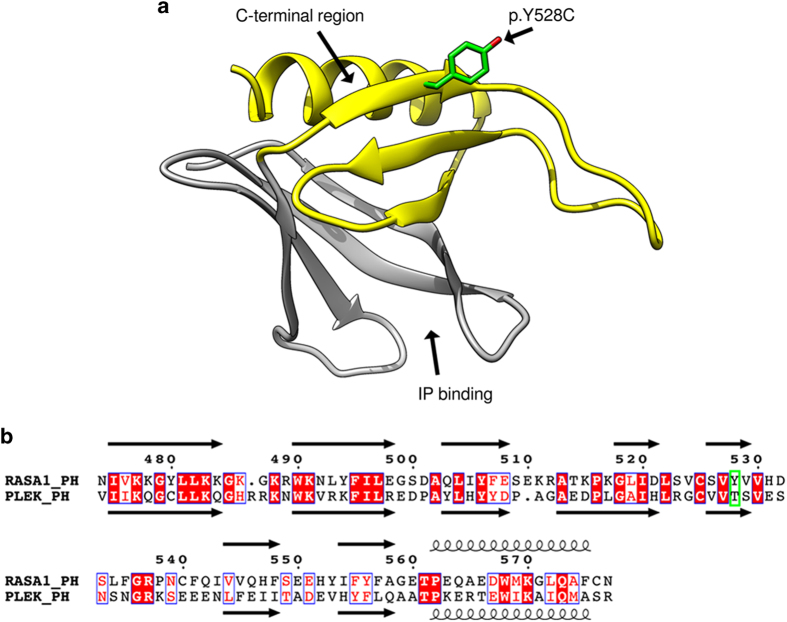
Structural analysis of the RASA1 PH domain detailing the impact of the identified p.Y528C (c.1583A>G) mutation. (**a**) The structure of the RASA1 PH domain is shown. The location of the variant p.Y528C is indicated in green, the inositol-phosphate (IP)-binding site is indicated and the C-terminal protein interaction domain is highlighted in yellow. (**b**). Sequence alignment between PH domain of RASA1 (RASA1_PH: residue 474–577) and PH domain of Pleckstrin (PLEK_PH: residues 235–347). Secondary structure obtained from RASA1_PH model and PLEK_PH (PDB:1XX0) are shown where arrows represent beta-sheet structure and curls represent alpha-helices. Conserved residues are white with red background, residues with similar properties are red with white background and the location of the p.Y528C variant is in a green box. The RASA1_PH domain has overall 30% identity with PLEX_PH domain with most identical residues present in secondary structure elements.

*RASA1-*related disorders follow an autosomal dominant pattern of disease inheritance and mutations in *RASA1* result in a number of syndromes such as CM–AVM syndrome, Parkes Weber syndrome and 5q14.3 neurocutaneous syndrome.^4^ The *RASA1* gene encodes p120RasGAP, which functions as an activator of non-oncogenic RAS-p21 GTPase activity. The domain structure of p120RasGAP consists of two SH2 domains split by an SH3 domain, a PH domain, a calcium-binding C2 domain and a C-terminal GAP domain, which modulates the activity of non-oncogenic RAS (Figure 3).^16,17^ The c.1583A>G (p.Y528C) missense variant identified in our cohort has been described previously in a family with CMs.^3^ In this study, the variant co-segregated with all three affected family members and is considered a pathogenic mutation. The p.Y528C mutation lies in the PH domain of the protein, which are structurally conserved but functionally diverse with many members binding phospholipids and their head group derivatives as well as being involved in several protein–protein interactions.^18^ The structure of the RASA1 PH domain resembles a barrel that consists of a seven stranded antiparallel beta-sheet, closed on one end by a C-terminal alpha-helix (Figure 4a, b).

Using literature on the RASA1 PH domain and PH domains in general, we were able to annotate the phospholipid-binding region and protein interaction region on the structure (Figure 4a,b).^16,18,19^ The C-terminal half of the PH domain (*RASA1* residues 523–591) has been shown to interact and compete with RAS for binding to its own catalytic GAP domain.^16^ The Tyr528 (p.Y528) side chain is surface exposed on the C-terminal half (residues 523–577) of the PH domain adjacent to the C-terminal alpha-helix (Figure 4a, b). The p.Y528C mutation would remove the aromatic side chain and introduce a smaller and slightly negatively charged residue. The surface exposed position of the amino acid indicates the mutation might alter binding of the PH domain to a partner protein such as the membrane-associated scaffolding protein RACK1 or its own GAP domain.^16,18,19^ Although the lack of structural data precludes us from interpreting the direct mutational effect, the p.Y528C mutation likely alters these binding interfaces and their important regulatory features rendering the GAP domain incapable of binding activated RAS.

**Figure 5:**
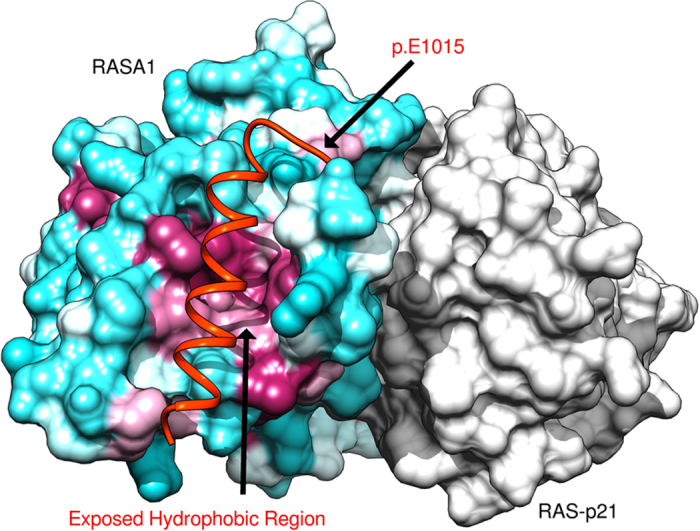
Graphical representation of RASA1 Ras-Gap domain and Ras-p21 crystal structure detailing the impact of the identified p.E1015* (c.3043G>T) mutation. The surface of RASA1 Ras-Gap domain (residues 718–1037) surface is colored based on the Kyte–Doolittle hydrophobicity scale (hydrophobic regions in purple and hydrophilic areas in turquoise) and the Ras-p21 is in shown with gray surface. The p.E1015* variant would remove the C-terminal helix (orange) and would act to destabilize the GTPase-activating protein (GAP) domain by exposing the hydrophobic region to solvent. Images generated with chimera.^14^

The lack of symptoms in the patient with the *RASA1* p.E1015* (c.3043G>T) nonsense mutation outside of pregnancy may be explained through a structural interpretation of the altered protein. The C-terminal region of RASA1 contains the GAP domain that is responsible for binding RAS-p21 and modulating its activity. The p.E1015* variant causes the loss of the terminal 32 amino acids of the RASA1 protein and the terminal helix in the Gap domain (Figure 5).^20^ Visual analysis of the structure shows that the terminal helix stabilizes part of the GAP domain and buries part of the core hydrophobic region (Figure 5, magenta color). As the majority of the GAP domain is intact, including the RAS-p21-binding surface, the p.E1015* truncation would only partially destabilize the domain. The extent of a destabilized GAP domain would ultimately determine the interaction between GAP and RAS-p21.

The p.E1015* nonsense mutation within the GAP domain may be further destabilized by a hormonal or placental affect preventing the GAP and RAS-p21 interaction, which would explain the onset of symptoms during pregnancy. The presentation of symptoms solely during pregnancy was similarly seen in another study where the patient harbored a deletion mutation (c.2927del) in the latter part of the RASA1 protein as well, which resulted in a frame shift.^21^ The patient tested negative for mutations in *ENG*, *ACVRL1* and *SMAD4*. Interestingly, the authors noted that the patient had features not typically associated with CM–AVM such as pulmonary capillary level microvascular shunting, similar to our described patient. With respect to HHT, it has been well documented that AVMs can be worse during pregnancy and may be due to hormonal factors.^22,23^

Our analysis also revealed a c.950G>A (p.R317Q) missense variant of unknown significance in *GDF2*. Predicted structural analysis of the variant revealed a possible role in disease. GDF2, growth differentiation factor 2, acts to stimulate transforming growth factor beta signaling pathway through interacting with the receptors ENG and ACVRL.^5^ The protein is synthesized as an inactive precursor that matures through cleavage and formation of a heterodimer between the N-terminal pro-region and C-terminal mature region.^24,25^ Immature GDF2 is cleaved by the furin protease that recognizes the general motif R^P4^- x^P3^—[R/K]^P2^—R^P1^ (where the letters are single amino acids, x represents any small amino acid and the positions are represented by superscript P4–P1, respectively) in which GDF2 contains the motif R316-R317-K318-R319.^25^ The c.950G>A variant overlaps the P3 position of the furin recognition sequence. Structural analysis reveals the furin catalytic domain is largely negatively charged, which supports a positively charged GDF2 peptide and its basic amino acid (p.R317) in position P3.^25^ The more neutral p.Q317 would not contribute the electrostatic stability provided by p.R317, decreasing GDF2 binding to furin for cleavage. Similarly, in a furin peptide cleavage assay, the presence of Q in the P3 position of the canonical furin motif was found to vastly minimize cleavage.^26^ The mutation is predicted to decrease the efficiency of furin cleavage altering levels of mature GDF2 and negatively affecting cellular signaling.

An analysis of the clinical features of the three subjects with mutations in either *RASA1* or *GDF2* was performed after molecular testing. A diagnosis of HHT still remains likely in individuals meeting at least 3 Curacao criteria and carrying a mutation in *GDF2*. Conversely, those with *RASA1* mutations who meet fewer than 3 Curacao criteria may need to be re-evaluated for a more plausible diagnosis such as a CM–AVM. Performing co-segregation analysis on affected family members, especially for the GDF2 p.R317Q variant of unknown significance, will aid in providing additional evidence in the classification of variants. It is important to note that although expert and comprehensive clinical evaluation can typically distinguish HHT, internal data suggest that significantly fewer patients are testing positive through molecular analysis than in well phenotyped cohorts. This suggests that, in current practice, clinical phenotyping may be incomplete or insufficient due to a delay in the presentation of symptoms or perhaps limited specialized clinical acumen.

In conclusion, our results suggest there is sufficient evidence that molecular screening of *RASA1* and *GDF2* will help clinicians provide a more accurate diagnosis for patients presenting with symptoms of HHT who test negative for mutations in *ENG*, *ACVRL1* and *SMAD4.*

## Figures and Tables

**Table 1:** Variants detected after filtering in *RASA1* and *GDF2* genes in patients suspected to have HHT

*Gene*	*Mutation*	*Coding variant*	*Protein*	*POLYPHEN prediction*	*SIFT prediction*	*Classification*
*RASA1*	Missense	c.1583A>G	p.Y528C	Probably damaging	Damaging	Likely pathogenic
*RASA1*	Nonsense	c.3043G>T	p.E1015^a^	Damaging	Damaging	Pathogenic
*GDF2*	Missense	c.950G>A	p.R317Q	Benign	Damaging	Unknown significance

Abbreviation: HHT, hereditary hemorrhagic telangiectasia.

a Premature stop codon (nonsense mutation).
